# High relative air humidity influences mineral accumulation and growth in iron deficient soybean plants

**DOI:** 10.3389/fpls.2014.00726

**Published:** 2014-12-18

**Authors:** Mariana Roriz, Susana M. P. Carvalho, Marta W. Vasconcelos

**Affiliations:** ^1^CBQF - Centro de Biotecnologia e Química Fina - Laboratório Associado, Escola Superior de Biotecnologia, Universidade Católica Portuguesa/PortoPorto, Portugal; ^2^Horticulture and Product Physiology Group, Department of Plant Sciences, Wageningen UniversityWageningen, Netherlands

**Keywords:** IDC, iron, mineral nutrition, RH, soybean

## Abstract

Iron (Fe) deficiency chlorosis (IDC) in soybean results in severe yield losses. Cultivar selection is the most commonly used strategy to avoid IDC but there is a clear interaction between genotype and the environment; therefore, the search for quick and reliable tools to control this nutrient deficiency is essential. Several studies showed that relative humidity (RH) may influence the long distance transport of mineral elements and the nutrient status of plants. Thus, we decided to analyze the response of an “Fe-efficient” (EF) and an “Fe-inefficient” (INF) soybean accession grown under Fe-sufficient and deficient conditions under low (60%) and high (90%) RH, evaluating morphological, and physiological parameters. Furthermore, the mineral content of different plant organs was analyzed. Our results showed beneficial effects of high RH in alleviating IDC symptoms as seen by increased SPAD values, higher plant dry weight (DW), increased plant height, root length, and leaf area. This positive effect of RH in reducing IDC symptoms was more pronounced in the EF accession. Also, Fe content in the different plant organs of the EF accession grown under deficient conditions increased with RH. The lower partitioning of Fe to roots and stems of the EF accessions relative to dry matter also supported our hypothesis, suggesting a greater capacity of this accession in Fe translocation to the aerial parts under Fe deficient conditions, when grown under high RH.

## Introduction

Humans and plants need essential micronutrients for the proper functioning of cellular processes (O'Rourke et al., 2009). Iron (Fe), in particular, is associated with serious cases of anemia, especially prevalent in developing countries, and in these countries plants constitute the major source of dietary Fe (Welch and Graham, 2004; Grotz and Guerinot, 2006). Despite iron's abundance in most soils, poor soil aeration, alkaline pH, high levels of bicarbonate, and root damage make it less available for absorption by plants (Celik et al., 2010).

Iron deficiency chlorosis (IDC) is a common symptom caused by lack of available Fe, occurring worldwide. It is characterized by a significant decrease in chlorophyll leaf content (Vasconcelos and Grusak, 2014), reduced leaf area and total plant dry weight (DW) (Zaharieva et al., 2004; Rabhi et al., 2007; Zocchi et al., 2007), resulting in diminished yield and crop quality (Vasconcelos and Grusak, 2014). Moreover, the Fe concentration in seeds and other plant organs is reduced in plants affected by IDC (Grusak, 1999). However, there are different susceptibilities to Fe deficiency between cultivars of the same species, and environmental conditions and genetically determined factors affect cultivar response to IDC (Vasconcelos and Grusak, 2014). Depending on the type of response to Fe deficiency, plants can be classified as “Fe-efficient” (EF) if they respond to Fe-deficiency by inducing biochemical reactions that make Fe available in a useful form and “Fe-inefficient” (INF) if they do not (Brown and Jolley, 1989).

Soybean (*Glycine max* L.) is one of the most highly produced legumes worldwide (Vasconcelos and Grusak, 2014) but is particularly susceptible to IDC. Several strategies for IDC control such as Fe foliar applications (Zuo and Zhang, 2011; King et al., 2013), Fe seed treatments (Karkosh et al., 1988; Goos and Johnson, 2001; Wiersma, 2005; King et al., 2013) and increasing seed density (King et al., 2013) have been tested with limited success. Until now, cultivar selection remains the most practical solution to overcome this nutrient deficiency (Goos and Johnson, 2001). Although some progress has been made in developing cultivars with enhanced tolerance to IDC, this effort has been hampered due to large environmental effects that interfere with the effectiveness of EF cultivars (Genotype × Environment interaction).

Besides the aforementioned factors, relative humidity (RH) has an important role on plant nutritional status. It is known that plants exposed to high RH might show increased growth due to higher stomatal opening, leading to increased uptake of CO_2_ (Gislerød and Nelson, 1989; Mortensen and Gislerød, 1990). This regulation is mediated by stomata and is directly related with the vapor pressure deficit (VPD) in the atmosphere. Stomatal conductance (g_s_) can indirectly provide valuable information about nutrient translocation in plants. It is known that the reduced transpiration due to increased RH (Ehret and Ho, 1986; Gislerød et al., 1987) diminishes the rate of xylem volume flow and therefore lowers nutrient translocation (White, 2012).

Low transpirational rates were found in chlorotic leaves because of the decrease in stomatal opening (Fernández et al., 2008). A study carried out by Eichert et al. (2010), carried out with peach trees, showed that Fe deficiency chlorosis limited xylem conductivity by reducing the size of xylem vessels, which may have an impact on the hydraulic potential. Torre et al. (2001) found that the xylem flow is mainly directed to the highly transpiring leaves, and that high transpirational rate, resulting from low RH, improves uptake and translocation of minerals in plants. On the contrary, in a study with *Citrus aurantium* L., Basiouny and Biggs (1971) reported that plants exposed to high RH levels showed higher Fe uptake rates. Thus, nutrient uptake is related to ambient RH and leaf transpiration hence affecting photosynthesis and nutrient transport (Tibbitts, 1979). However, the explanations for these responses are contradictory.

Taking into account the importance of RH in the plant's nutritional status, we decided to study the effect of exposure of two accessions with different IDC tolerances, grown under Fe-sufficient (SUF) and Fe-deficient (DEF) conditions, to low (60%) and high (90%) RH. Plants were scored based on the degree of leaf chlorosis (SPAD measurements) and several morphological and physiological parameters such as total plant DW, plant height, root length, shoot DW, root:shoot ratio, and leaf area were measured. We also analyzed the mineral content on the different plant organs (roots, stems, unifoliate and trifoliate leaves) and calculated the partition quotient (PQ), to evaluate the effect of high RH in the flow of several minerals (Fe, Cu, Mn, B, Mg, Zn, Ca, Mo, P, and K) involved in plant growth and the role of RH on mineral distribution. Ultimately, we were able to relate the degree of plant IDC symptoms with its nutritional status, as affected by RH.

## Materials and methods

### Plant material and growth conditions

Two *G. max* accessions with differential IDC response were obtained from the USDA (U. S. Department of Agriculture) germplasm collection via GRIN (Germplasm Resources Information Network) (http://www.ars-grin.gov/): an EF accession - PI 360952, “Amurskaja 310” and an INF accession - PI 407707, “Chuen sien No. 1.” These accessions have contrasting efficiencies in Fe absorption based on the study of Vasconcelos and Grusak (2014). Plants were cultivated in two greenhouses under a 20-h light/4-h darkness photoperiod at 21°C. Two RH conditions were used: low (60%) and high (90%) and the photon flux density during the day was about 287 μmol m^−2^ s^−1^, supplied with fluorescent lamps. In order to stimulate germination, a cut was made in seeds of *G. max* with a razor blade and germination was achieved in wet filter paper supplemented with 250 mM CaCl_2_ for 7 days in the dark, before transfer to hydroponic solution with different Fe treatments. The standard solution for hydroponically grown plants contained: 1.2 mM KNO_3_; 0.8 mM Ca(NO_3_)_2_; 0.3 mM MgSO_4_.7H_2_O; 0.2 mM NH_4_H_2_PO_4_; 25 μM CaCl_2_; 25 μM H_3_BO_3_; 0.5 μM MnSO_4_; 2 μM ZnSO_4_.H_2_O; 0.5 μM CuSO_4_.H_2_O; 0.5 μM MoO_3_; 0.1 μM NiSO_4_. All nutrients were buffered with 1 mM MES (2,4-morpholino-ethane sulfonic acid) (pH 5.5). The solutions were supplemented with 5 μM Fe(III)-EDDHA [ethylenediamine-N,N′bis(o-hydroxyphenyl) acetic acid] for 7 days followed by 0 μM for an additional 7 days (DEF conditions) or with 20 μM for 14 days (SUF conditions), the latter being replaced every 2 days.

### SPAD measurements

Leaf chlorophyll content was assessed for evaluation of plant tolerance or susceptibility to Fe deficiency chlorosis. A portable SPAD 502-Plus Chlorophyll Meter (Minolta corporation, Ltd., Osaka, Japan) was used for estimating the chlorophyll concentration (Lima et al., 2014). Three SPAD readings were taken in the youngest trifoliate leaves around the midpoint of each leaf of five plants per accession.

### Stomatal conductance

Leaf stomatal conductances were measured in unifoliate leaves, 2.5 h after switching on the lights, with a AP4 Leaf Porometer (Delta-T Devices, UK), in order to evaluate the impact of Fe deficiency and RH on plant-atmosphere water exchange. Three g_s_ measurements were done in one of the unifoliate leaves of five plants per accession.

### Morphological traits

To study the effect of RH on plant growth, a series of morphological traits were evaluated. Thus, five plant parts were discerned: roots, stems, unifoliate and trifoliate leaves. After drying tissues at 70°C for 2 days, DW was recorded and the root:plant ratio was calculated as the ratio between the DW of roots and the DW of the whole plant. Plant height and root length were also measured. An AM300 Portable Leaf Area Meter (ADC Bioscientific Ltd., UK) was used for determining total leaf area. Five plants per accession were used to study morphological parameters.

### Microwave-assisted digestion and ICP analysis

Two hundred mg of the different dried plant organs (roots, stems, unifoliate and trifoliate leaves) of the two *G. max* accessions (EF - PI 360952 and INF - PI 407707) were mixed with five mL of 65% HNO_3_ in a Teflon reaction vessel and heated in a SpeedwaveTM MWS-3+ (Berghof, Germany) microwave system. Digestion procedure was conducted in five steps: 1-130°C/10 min, 2-160°C/15 min, 3-170°C/12 min, 4-100°C/7 min, 5-100°C/3 min. The resulting clear solutions of the digestion procedure were then brought to 20 mL with ultrapure water for further analysis. A bulked sample of five plants per accession was used for digestion. Nutrient content was analyzed in the plant organs described above by ICP-OES Optima 7000 DV (PerkinElmer, USA). All analysis was done in triplicate.

### Partition quotient calculation

To evaluate the partitioning of minerals within *G. max* plants grown under DEF conditions, changes in each tissue's content were normalized to changes in each tissue's weight, relative to the whole plant. The DW of each organ was calculated as a percentage of total plant DW at each RH, and mineral content of each organ was calculated as a percentage of total plant mineral content at each RH. Using these values, the normalized partitioning of each mineral within the plant was calculated by dividing each organ's percentage mineral content by its percentage DW, and multiplying by 100, which we refer to as the partition quotient, as described by Waters and Grusak (2008).

### Statistical analysis

Significant differences between treatments and cultivars were determined using the unpaired Student's *t*-test corrected for multiple comparisons using the Holm-Sidak method. All statistical analyzes were carried out using GraphPad Prism version 6.00 for Mac OS X (GraphPad Software, Inc., La Jolla, CA, USA). Statistical significance was considered at *P* < 0.05.

## Results

### Characterization of morphological and physiological parameters

**Figure 1:**
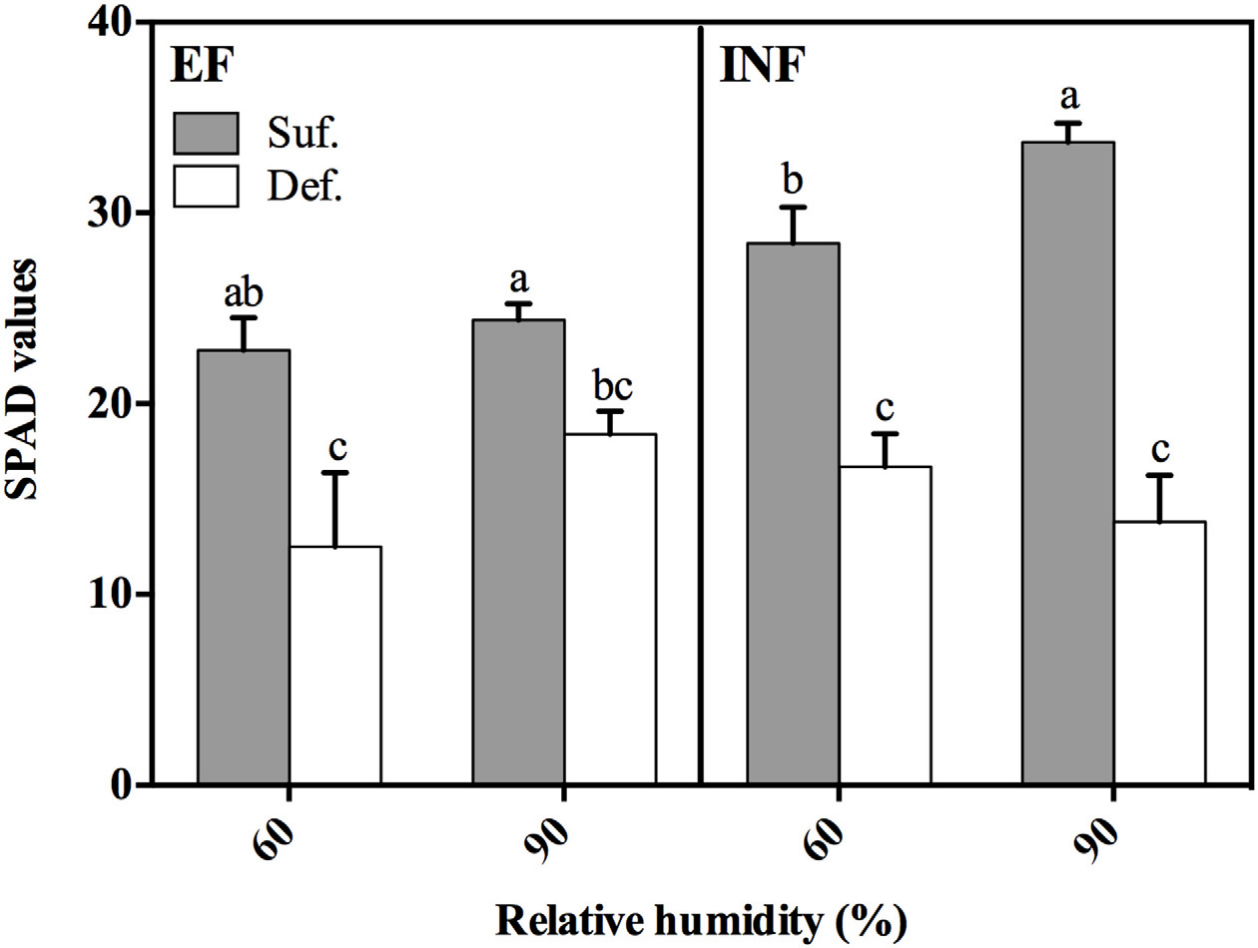
**SPAD values measured at 14 days after transferal to hydroponic conditions in “Fe-efficient” (EF, PI 360952) and “Fe-inefficient” (INF, PI 407707) accessions and grown at 60% and 90% relative humidity (RH) under Fe-sufficient (SUF) and Fe-deficient (DEF) conditions**. Data are Means ± SEM. Different letters indicate significant differences (*P* < 0.05) between treatments.

**Figure 2:**
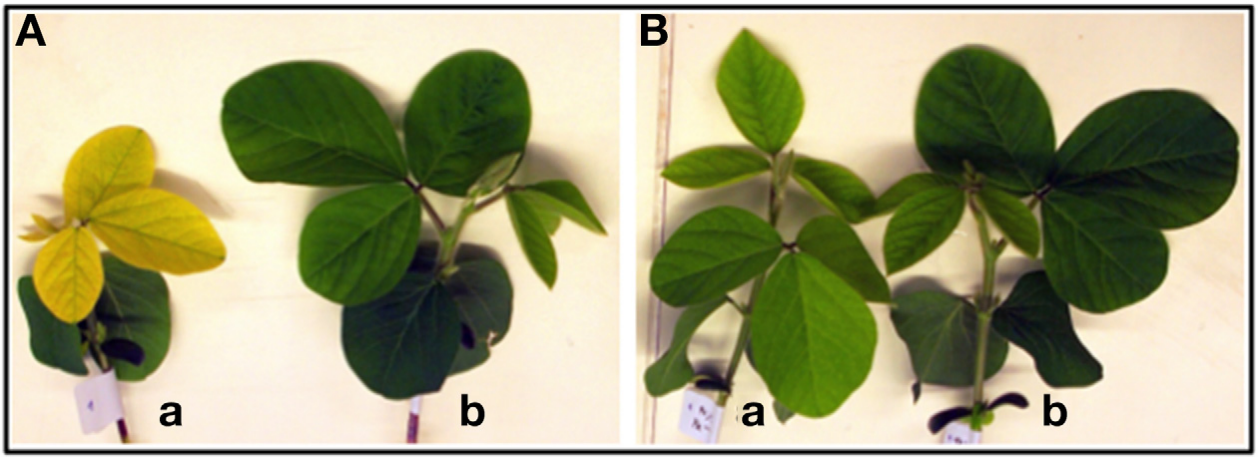
**Fourteen-day-old shoots of “Fe-efficient” (EF) accession (PI 360952) grown under Fe-deficient (DEF) (a) and Fe-sufficient (SUF) (b) conditions at 60% (A) and 90% (B) relative humidity (RH)**.

The impact of Fe deficiency was more pronounced in plants of the EF accession grown at 60% RH which developed the typical IDC symptoms: stunted growth and a decreasing chlorophyll concentration trend (SPAD values 32% lower) relative to plants grown under high RH (Figures 1, 2). There was only a significant increase of 16% in SPAD values of plants grown under Fe sufficient conditions with RH (Figure 1).

In the EF plants grown under Fe deficient conditions, we found that the increase in RH resulted in significantly increased plant height (25%), root length (33%), plant DW (42%), and leaf area (39%) but it had no significant effect on the root:shoot ratio (Table 1). Regarding the INF accession, with exception of the root:shoot ratio which was significantly higher (39%), there was no significant effect of high RH in plants grown under Fe deficient conditions, although there was a tendency for an increase in all measured parameters (Table 1).

**Table 1:** **Plant growth of “Fe-efficient” (EF, PI 360952) and “Fe-inefficient” (INF, PI 407707) *Glycine max* (*G. max*) accessions measured 14 days after transfer to hydroponic conditions grown at 60% and 90% relative humidity (RH) under Fe-sufficient (SUF) and Fe-deficient (DEF) conditions (Fe supply)**.

***G. max* accession**	**RH (%)**	**Fe supply**	**Plant height (cm)**	**Root length (cm)**	**Plant DW (g)**	**Root:shoot ratio (g.g^−1^)**	**Leaf area (cm^2^)**
EF	60	SUF	10.7 ± 0.6^ab^	39.5 ± 1.4^a^	0.87 ± 0.07^a^	0.32 ± 0.01^a^	151.3 ± 13.3^a^
		DEF	8.7 ± 0.4^a^	25.3 ± 0.2^b^	0.43 ± 0.03^b^	0.36 ± 0.02^ab^	75.0 ± 19.6^b^
	90	SUF	12.4 ± 0.4^b^	37.6 ± 1.8^a^	0.89 ± 0.04^a^	0.37 ± 0.01^b^	147.9 ± 2.9^a^
		DEF	11.6 ± 0.6^b^	37.5 ± 2.2^a^	0.74 ± 0.06^a^	0.41 ± 0.02^b^	123.5 ± 5.0^c^
INF	60	SUF	10.8 ± 0.5^ac^	45.4 ± 0.3^a^	0.86 ± 0.26^a^	0.34 ± 0.01^ab^	202.3 ± 13.9^a^
		DEF	8.5 ± 0.8^b^	47.7 ± 3.7^ab^	0.36 ± 0.03^b^	0.20 ± 0.05^a^	42.9 ± 14.1^b^
	90	SUF	12.1 ± 0.5^a^	54.2 ± 6.5^ab^	1.19 ± 0.03^a^	0.32 ± 0.03^ab^	1409.0 ± 183.5^c^
		DEF	10.0 ± 0.6^bc^	54.1 ± 1.3^b^	0.51 ± 0.06^b^	0.33 ± 0.02^b^	72.2 ± 17.8^b^

*Data are Means ± SEM. Different letters indicate significant differences (P < 0.05) between treatments. Statistical analysis was performed independently for each accession*.

Concerning the morphological changes induced by Fe deficiency, we did not find significant differences for plant height and root:shoot ratio between the two studied soybean accessions, in all treatments (Table 1) (statistical analysis performed separately for this comparison). However, roots of the INF plants grown under deficiency were significantly longer (Table 1).

Dry weight of INF plants grown under Fe sufficient conditions was significantly higher when plants were grown under Fe sufficiency (Table 1). The leaf area of the EF plants significantly increased under Fe sufficient conditions at both 60 and 90% humidity (Table 1).

**Figure 3:**
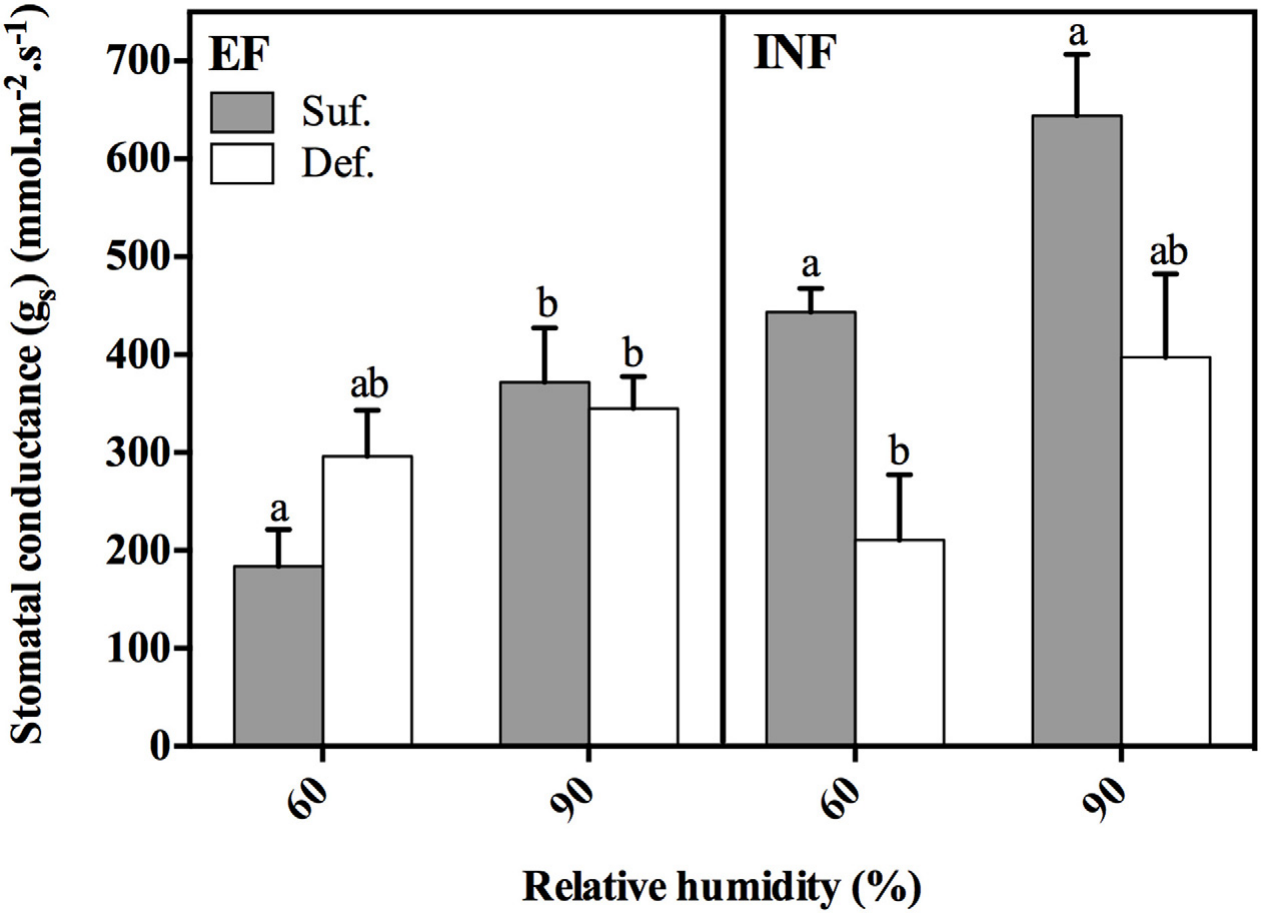
**Stomatal conductance (g_s_) measured in the 14-day-old unifoliate leaves, 2 h 30 min after the lights were switched on for each *Glycine max* (*G. max*) accession (“Fe-efficient,” EF, PI 360952 and “Fe-inefficient,” INF, PI 407707) grown at 60% and 90% relative humidity (RH) under Fe-sufficient (SUF) and Fe-deficient (DEF) conditions**. Data are Means ± SEM. Different letters indicate significant differences (*P* < 0.05) between treatments. Statistical analysis was performed independently for each accession.

For both accessions, there was a tendency for the increase of g_s_ with RH regardless of Fe status, but it was only statistically significant for the EF accession grown under sufficient conditions (Figure 3). In the EF plants, the limitation of Fe in solution seemed to produce plants with increased g_s_ when grown at 60% RH and slightly decreased g_s_ when grown at 90% RH, although these differences were not statistically significant (P > 0.05). For the INF accession, the lack of Fe significantly decreased (52%) the leaf g_s_ at low RH. Also, g_s_ of INF plants grown under Fe sufficient conditions, at both RH conditions, was significantly higher comparatively to that obtained by the EF plants (statistical analysis performed separately for this comparison).

### Plant mineral content

**Figure 4:**
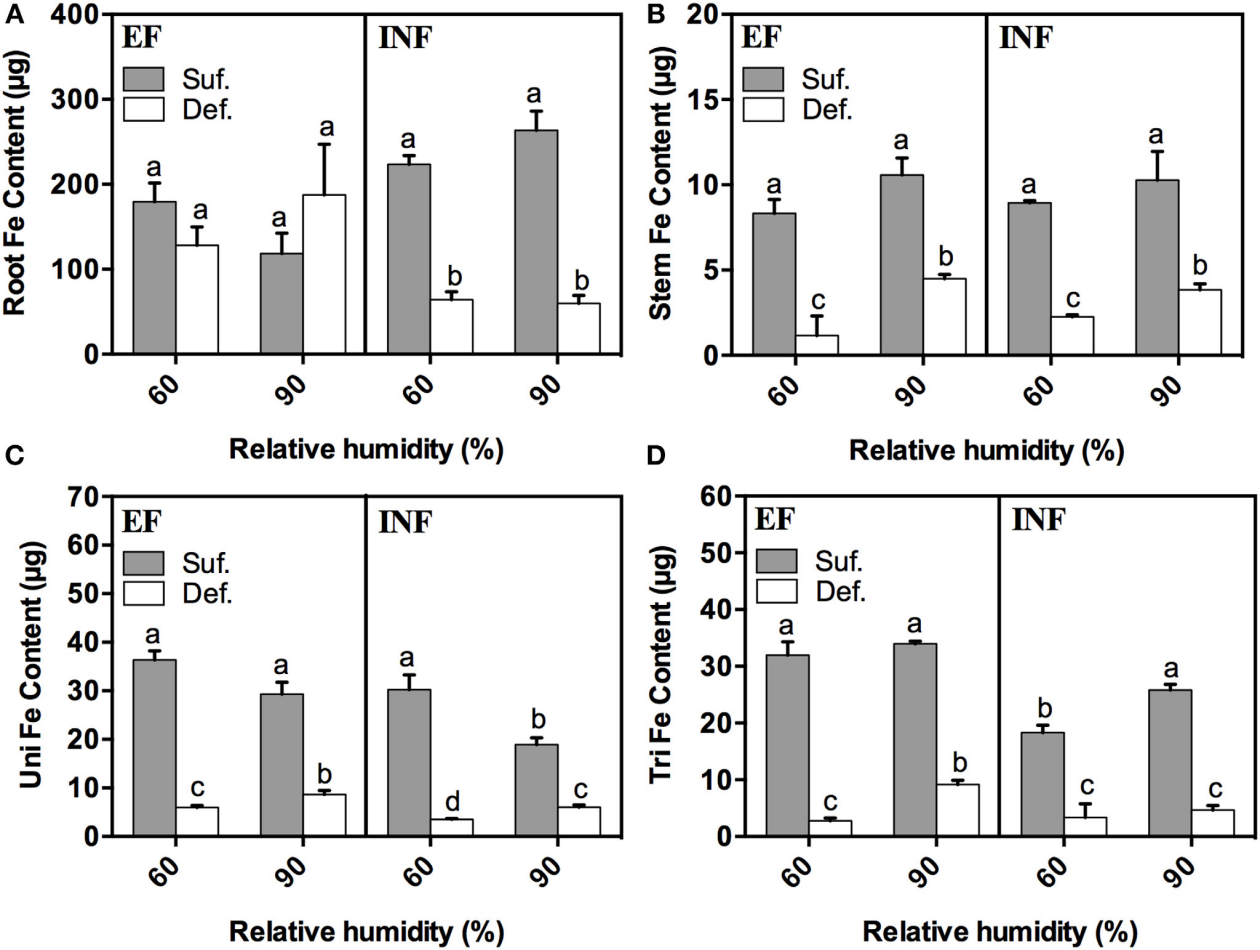
**Fe content (μg) measured by ICP-OES in 14-day-old roots (A), stems (B), unifoliate (Uni) (C), and trifoliate (Tri) leaves (D) of “Fe-efficient” (EF, PI 360952) and “Fe-inefficient” (INF, PI 407707) accessions and grown at 60% and 90% relative humidity (RH) under Fe-sufficient (SUF) and Fe-deficient (DEF) conditions**. Data are means ± SEM. Different letters indicate significant differences (*P* < 0.05) between treatments. Statistical analysis was performed independently for each accession.

RH had a significant impact on mineral distribution and accumulation in the different plant organs. Therefore, we decided to evaluate the content of a series of minerals in different plant organs, giving particular emphasis to Fe. In general, for all treatments, the Fe content was significantly lower in plants under deficiency grown at 60% and 90% RH in every studied plant organ (Figures 4A–D). The sole exception was for the roots of the EF plants grown at both humidity levels. The highest Fe content was found in roots (Figure 4A) and the lowest in stems (Figure 4B). We could see that, in roots, there was a significant decrease in the Fe content of the INF accession grown under Fe deficient conditions, at both RH conditions, comparatively to the EF accession where the high RH seemed to increase (32%) (*P* > 0.05) the Fe content in roots of plants grown under Fe deficiency (Figure 4A). The Fe content in stems and in unifoliate leaves significantly increased with RH in plants grown under Fe limitation for both accessions (Figures 4B,C). This was also verified in the trifoliate leaves of the EF accession (Figure 4D).

### Partition quotient analysis

A PQ value, representing the proportional mineral content in an organ relative to the proportional DW of the organ, was calculated to allow comparison of the dynamics of partitioning of minerals between the different plant organs when grown under DEF conditions and to check if the minerals content is influenced by RH.

**Figure 5:**
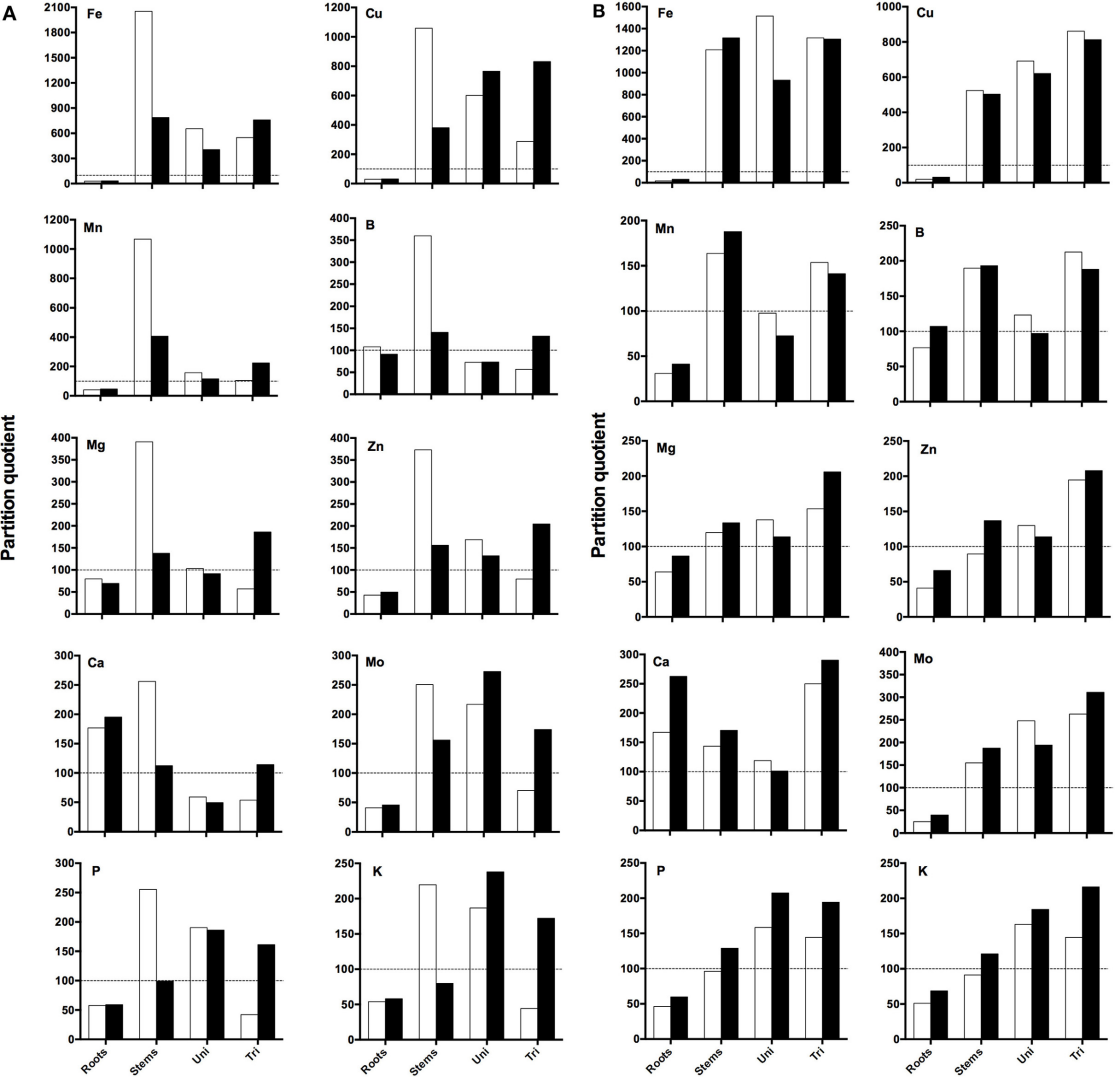
**(A)** Partition quotients (PQ) of Fe, Cu, Mn, B, Mg, Zn, Ca, Mo, P, and K in roots, stems, cotyledons (Cot), unifoliate (Uni), and trifoliate (Tri) leaves of “Fe-efficient” (EF) accession grown under Fe-deficient (DEF) conditions at 60% (open bars) and 90% (closed bars) relative humidity (RH). Y-axis range varies among elements. Dashed horizontal line represents PQ of 100 (the percentage contribution of the organ to the plant dry weight (DW) is the same as the percentage contribution to the plant's total content of the mineral being evaluated). **(B)** Partition quotients (PQ) of Fe, Cu, Mn, B, Mg, Zn, Ca, Mo, P, and K in roots, stems, cotyledons (Cot), unifoliate (Uni), and trifoliate (Tri) leaves of “Fe-inefficient” (INF) accession grown under Fe-deficient (DEF) conditions at 60% (open bars) and 90% (closed bars) relative humidity (RH). Y-axis range varies among elements. Dashed horizontal line represents PQ of 100 (the percentage contribution of the organ to the plant dry weight (DW) is the same as the percentage contribution to the plant's total content of the mineral being evaluated).

When looking at the EF plants (Figure 5A) it can be seen that high RH decreased PQ values in roots and stems and increased PQ values in unifoliate and trifoliate leaves. Roots presented the lowest PQ values, reaching over 100 only for Ca at both RH conditions (Ca showed the highest PQ values in roots) and for B at 60% RH; here RH didn't influence the PQ values. Stems were the organs that presented the highest PQ values at 60%, except for Mo and K; PQ values of Fe, Mn, Mg, Zn, and Ca in unifoliate leaves decreased with the increase in RH. PQ values of trifoliate leaves increased for every mineral at 90% RH. The PQ expression pattern was similar for Fe, Mn, Mg, and Zn, although the order of magnitude was different; Fe was the mineral that presented the highest PQ values of all studied minerals.

When looking at the INF plants (Figure 5B), contrary to what was observed for the EF accession, PQ values of roots and stems increased at 90% RH. Once again, roots of the INF accession presented the lowest PQ values, reaching over 100 only for Ca at both RH conditions and for B at 90% RH, with Ca showing the highest PQ values in roots. In this case, trifoliate leaves presented the highest PQ values, except for Fe, Mn, and P. The PQ values in unifoliate leaves decreased at high RH except for K and P and increased at 90% RH in trifoliate leaves, except for Fe, Cu, Mn, and B. A similar PQ expression pattern was verified for Mn, Mg, Zn, Ca, and Mo although the order of magnitude was different. Once again, Fe showed the highest PQ values in the INF accession out of all studied minerals. PQ values of Fe and the other minerals (except Ca) from the INF accession were lower than those from the EF accession.

## Discussion

Plant transpiration, among other factors, influences the flux of minerals and water between roots and shoots (Bouranis et al., 2014). To the best of our knowledge this is the first study on the characterization of the impact of high RH in the response of two soybean cultivars with different IDC susceptibilities to Fe deficiency.

### Characterization of plant morphological and physiological parameters

The leaf chlorophyll content measured through SPAD values helps in the evaluation of plant IDC degree. We concluded that the high RH produced a more positive effect in the EF accession, resulting in plants with reduced IDC symptoms. In fact, when these plants were grown under DEF conditions, the high RH produced plants with increased chlorophyll content (SPAD values) comparatively to plants grown at low RH (Figure 1). However, this increase was not verified in INF accession: SPAD values decreased 17% in plants grown under DEF conditions with the increase in RH (Figure 1). The beneficial effect exerted by the exposure of EF accession to high RH can be explained by their ability to better mobilize the Fe. Thus, we note that high RH did not improve IDC response in INF accession, but there is a clear positive effect for plants which have already the machinery to respond more efficiently to Fe limitation.

The beneficial effect that high RH produced in suppressing IDC symptoms of the EF accession could also be seen through the other analyzed morphological parameters. High RH exposure led to an increased plant height, root length, plant DW, and leaf area (Table 1) grown under Fe deficient conditions, indicating that these plants were more vigorous. The reason for this higher biomass production, even under stress conditions, might be related to a significantly increased leaf area (39%) (Table 1) and slightly higher gs (14%) (Figure 3). This increase in leaf area may have enhanced the light interception and the leaf photosynthetic rate. High RH has also been shown to increase shoot growth and shoot-to-root ratio in cotton plants (Hoffman and Rawlins, 1971). The increase in leaf area caused by high RH could be linked to differential epidermal cell expansion and consequently altered stomatal densities, as suggested by Murphy et al. (2013).

On the other hand, the root:shoot ratio of plants of the INF accession grown under Fe deficiency was significantly higher when RH increased because the INF accession invested on the production of higher root biomass, the organs responsible for the uptake of Fe, to overcome Fe deficiency. We can conclude that the different ability of the two soybean accessions for Fe absorption is linked to root length, plant DW, and leaf area but not to plant height, as Elmstrom and Howard (1969) found. In their study with an EF (Hawkeye) and an INF (PI-54619-5-1) soybean accession, they verified that the EF plants were the shortest.

Our results confirm that plant growth is affected by RH, as suggested before. Gislerød and Mortensen (1990) found that the DW, leaf size, plant diameter, and height of *Begonia* × *hiemalis* “Schwabenland Red” grown at high RH were significantly higher than those found in plants grown at low RH; the increase in leaf size as a result of exposure to high RH have been reported as well on lettuce, wheat, sugar beet, cotton, and numerous greenhouse plants by Hoffman and Rawlins (1971), Ford and Thorne (1974), Tibbitts and Bottenberg (1976), and Mortensen (1986); high RH also proved to increase shoot and petiole elongation, resulting in increased plant height (Mitchell and Hoff, 1977).

### Mineral quantification

Concerning the Fe content—our main focus—in all studied plant organs, it significantly decreased when plants were grown under Fe limiting conditions in both accessions, regardless of RH (Figures 4A–D), as expected. Fernández et al. (2008) also showed, in a study with pear and peach that the Fe concentration in leaves was lower in plants grown under Fe deficient conditions. These results are in accordance with the lowest SPAD values found in plants grown under Fe limitation (Figure 1), suggesting that higher concentrations of Fe in trifoliate leaves (where SPAD readings were performed) implies higher IDC tolerance, as Fe is essential for the photosynthetic processes and chlorophyll production (Zheng, 2010). The highest Fe content found in roots (Figure 4A) is expected, as roots are the primary organs of Fe absorption. As suggested by Legay et al. (2012) perhaps plants have the ability to limit Fe transport to shoots and store it in roots. But it should be noted that part of this Fe was likely stored as precipitates in the root free space (apoplasmic accumulation), and may account for part of the total Fe determined in roots (Bienfait et al., 1985; Vert et al., 2003) whose availability for absorption and translocation is unclear. Still, bean plants are usually able to mobilize this Fe pool when grown under Fe deficiency (Bienfait et al., 1985). The low Fe content found in plants of the INF accession grown under DEF conditions at high RH (Figure 4A), can be related with their diminished ability to efficiently uptake the Fe, comparatively to the EF accession. These results showed that high RH does not improve Fe uptake in the INF accession. Also, the high Fe content found in stems and unifoliate leaves of both accessions and in trifoliate leaves of the EF accession when plants were grown under Fe deficient conditions at high RH, also supports our theory about the positive effect that high RH has in the alleviation of IDC symptoms, in this case through the enhancement of Fe accumulation.

The calculation of PQ allowed us to better understand the effect of RH in the mineral distribution of plants subjected to Fe limitation. The low PQ values obtained in roots and stems and high in unifoliate and trifoliate leaves of the EF accession grown at high RH for almost every studied mineral (Figure 5A) are indicative that, under deficient conditions, the high RH induced these plants to invest more in the uptake of minerals in the organs responsible for nutrient absorption (roots) and in the organs responsible for the transport of nutrients from the roots to the shoots (stems), to withstand Fe deficiency and properly develop.

On the contrary, the low PQ values found in roots of both accessions for almost every mineral are associated with an increased remobilization of nutrients to the aerial parts, regardless of RH. The highest PQ values found in stems of EF accession may be related to the fact that they are responsible for the transport of minerals between the below ground organs to the aerial parts, and so, it is expected that here the mineral content is low, also shown by the results of the Fe content (Figure 4). PQ values increased in the EF trifoliate leaves of plants grown at 90% RH, indicating that these organs were well-developed, supporting the results obtained in the previous analysis. The highest PQ values were found for Fe, as expected, as Fe is deficient in solution, and thus, plants had to invest in absorbing more Fe than other minerals. By contrast, high PQ values were found in roots and stems of the INF accession grown at 90% RH. These high PQ values for Fe in the roots may have been influenced by apoplastic accumulation of Fe in the roots (Bienfait et al., 1985). The increase in root and stem biomass induced by high RH may facilitate nutrient uptake and distribution to the aerial parts. High RH also increased PQ values of trifoliate leaves of INF accession, leading plants to invest more in increasing trifoliate biomass probably because these organs are not so vigorous under deficient conditions, as those from the EF accession, or because plants need to improve the photosynthetic surface area. Once again, PQ values of roots were the lowest and Fe was the mineral with the highest PQ values, for the same reasons presented above.

These differences in the PQ values between treatments can also be associated with the mobility of minerals in the xylem because of the impact of RH on the transpiration rates. Fe is transported to the shoot through the transpiration stream, circulating in the xylem as ferric-citrate complexes (Rellán-Álvarez et al., 2010) and later on imported into the leaf cytoplasm. Eichert et al. (2010) showed that Fe deficiency chlorosis may limit xylem conductivity by reducing the size of xylem vessels. It is possible that our observed reduction in g_s_ by the Fe deficient IN plants (Figure 3) could be related to this phenomenon. Interestingly, the EF plants did not exhibit this abrupt reduction of g_s_ caused by Fe deficiency, which could partly explain the increased resistance to IDC.

Fe must also be transported through the phloem, because the transpiration flow in the xylem vessels is inefficient in apexes, seeds, and root tips (Kim and Guerinot, 2007). Minerals have different mobilities in the phloem (White, 2012), but the impact of RH on phloem mobility cannot be directly explained.

Finally, all the performed analysis allowed us to conclude, through morphological and physiological parameters, that effectively, high RH affected plant growth and development. Furthermore, high RH alleviated IDC symptoms in Fe efficient plants grown under deficient conditions. This mitigation could be seen through increased SPAD values (Figure 1), plant height, root length, plant DW, leaf area, and Fe content in every studied plant organ. Also, the low PQ values found in roots and stems of the EF accession and high in unifoliate and trifoliate leaves support our hypothesis.

## Author contributions

Mariana Roriz carried out the sample preparation and analysis, mineral quantification by ICP-OES and drafted the manuscript; Susana M. P. Carvalho helped conceive the study and its design, and participated in the critical review of the manuscript; Marta W. Vasconcelos conceived the study, its design and coordination and helped to draft the manuscript. All authors read and approved the final manuscript.

### Conflict of interest statement

The authors declare that the research was conducted in the absence of any commercial or financial relationships that could be construed as a potential conflict of interest.

## Abbreviations

DEF: “Fe-deficient”
DW: Dry Weight
EF: “Fe-efficient”
GRIN: Germplasm Resources Information Network
ICP-OES: Inductively Coupled Plasma Optical Emission Spectrometer
IDC: Iron Deficiency Chlorosis
INF: “Fe-inefficient”
RH: Relative Humidity
g_s_: Stomatal Conductance
SEM: Standard Error of Mean
SUF: “Fe-sufficient”
USDA: U.S. Department of Agriculture.
